# A Novel Personalized Systems Nutrition Program Improves Dietary Patterns, Lifestyle Behaviors and Health-Related Outcomes: Results from the Habit Study

**DOI:** 10.3390/nu13061763

**Published:** 2021-05-22

**Authors:** Iris M. de Hoogh, Barbara L. Winters, Kristin M. Nieman, Sabina Bijlsma, Tanja Krone, Tim J. van den Broek, Barbara D. Anderson, Martien P. M. Caspers, Joshua C. Anthony, Suzan Wopereis

**Affiliations:** 1 TNO, Netherlands Organization for Applied Scientific Research, 3704 HE Zeist, The Netherlands; iris.dehoogh@tno.nl (I.M.d.H.); sabina.bijlsma@tno.nl (S.B.); tanja.krone@tno.nl (T.K.); tim.vandenbroek@tno.nl (T.J.v.d.B.); martien.caspers@tno.nl (M.P.M.C.); 2 Winters Nutrition Associates, S Abington Township, PA 18411, USA; blwinters13@gmail.com; 3 Katalyses, LLC, Ankeny, IA 50023, USA; knieman@katalyses.com; 4 Independent Researcher, Elmhurst, IL 60126, USA; Barbara.Andersonrd@gmail.com; 5 Habit, Oakland, CA 94607, USA; josh@nlumn.com; 6 Campbell Soup Company, Camden, NJ 08103, USA

**Keywords:** personalized nutrition, healthy lifestyle, systems biology, dietary intervention, mixed meal tolerance test

## Abstract

Personalized nutrition may be more effective in changing lifestyle behaviors compared to population-based guidelines. This single-arm exploratory study evaluated the impact of a 10-week personalized systems nutrition (PSN) program on lifestyle behavior and health outcomes. Healthy men and women (*n* = 82) completed the trial. Individuals were grouped into seven diet types, for which phenotypic, genotypic and behavioral data were used to generate personalized recommendations. Behavior change guidance was also provided. The intervention reduced the intake of calories (−256.2 kcal; *p* < 0.0001), carbohydrates (−22.1 g; *p* < 0.0039), sugar (−13.0 g; *p* < 0.0001), total fat (−17.3 g; *p* < 0.0001), saturated fat (−5.9 g; *p* = 0.0003) and PUFA (−2.5 g; *p* = 0.0065). Additionally, BMI (−0.6 kg/m^2^; *p* < 0.0001), body fat (−1.2%; *p* = 0.0192) and hip circumference (−5.8 cm; *p* < 0.0001) were decreased after the intervention. In the subgroup with the lowest phenotypic flexibility, a measure of the body’s ability to adapt to environmental stressors, LDL (−0.44 mmol/L; *p* = 0.002) and total cholesterol (−0.49 mmol/L; *p* < 0.0001) were reduced after the intervention. This study shows that a PSN program in a workforce improves lifestyle habits and reduces body weight, BMI and other health-related outcomes. Health improvement was most pronounced in the compromised phenotypic flexibility subgroup, which indicates that a PSN program may be effective in targeting behavior change in health-compromised target groups.

## 1. Introduction

Public health dietary recommendations are designed to help a majority of the population avoid chronic disease. Personalization of these recommendations is limited to gender and age [1]. However, people also differ in genotype, phenotype, behavior, personality and socio-psychological environment. Due to these differences, personal variation in response to dietary recommendations is likely. Indeed, research has shown that responses to nutritional interventions depend on differences in both genotype and phenotype [2,3,4]. Tailoring advice based on individual data also increases the perceived relevance of this advice [5]. Additionally, awareness of potential health problems leads to more favorable attitudes toward personalized nutrition [6,7]. Workforce wellness programs appear to be more effective if the content is tailored to participants’ needs [8]. Thus, personalized nutrition approaches may be more effective in changing dietary and other lifestyle behaviors, ultimately improving health outcomes, as compared to guidelines derived for the majority of the population [9,10,11,12].

Personalized nutrition has been defined as “the use of individual-specific information, founded in evidence-based science, to promote dietary behavior change that may result in measurable health benefits” [13]. The effectiveness of personalized nutrition programs can be enhanced by using an integrated systems-based approach [14]. A four-step cycle of personalized nutrition was designed to improve and sustain health and function by combining objective health data and behavior change to meet individual needs and goals [13]. This cycle starts with collecting individual-specific information, which may range from an individual’s current lifestyle and personal preferences to phenotype and genotype. In general, the level of personalization is dependent on the robustness and extensiveness of the available data [15]. The second step in the cycle is to translate individual data into evidence-based dietary recommendations. This requires the identification of food-health relationships using scientific knowledge and/or algorithms that can link individual data to dietary advice. Furthermore, it requires integration with a person’s needs, context and preferences to promote understanding, adherence and sustained behavior change [16,17].

The third component of the personalized nutrition cycle is to further promote dietary behavior change through the application of behavior change techniques, such as goal-setting, self-monitoring and positive feedback, which have been proven to be effective in increasing the likelihood of behavior change [18,19,20,21]. It has been shown previously that combining multiple approaches unique to the individual, including face-to-face contact, increases effectiveness [22,23,24,25,26]. Personalized behavior change support should also take into account readiness and motivation to change [17]. Regarding goal-setting, intrinsic motivation to achieve the goal and freedom in choosing goals are important determinants for success, both in the short and long term [27,28,29].

The fourth component of the personalized nutrition cycle measures the success of the advice and behavior change support; quantifiable improvements in health are essential. As personalized nutrition approaches strive to become more individualized and holistic, measuring the effects of such interventions demands an outcome measure that considers multiple aspects of health. A holistic definition of health has been defined by Huber et al. as “the ability to adapt or cope with ever changing environmental conditions” [30,31]. The ability of the metabolic system to recognize an environmental challenge, respond, and return to homeostasis is referred to as phenotypic flexibility [32]. An unhealthy lifestyle is known to impair phenotypic flexibility and may negatively affect health [32]. For example, impaired phenotypic flexibility has been reported in overweight participants who have a reduced ability to metabolize stored lipids for energy synthesis and adapt more slowly to excess dietary fat intake, compared with lean participants [33]. Assessment of phenotypic flexibility can be used as a measure of metabolic health status to inform nutritional interventions [34,35,36]. The assessment of phenotypic flexibility requires the perturbation of homeostasis and subsequent evaluation of nutrition-related biomarkers. A nutrition challenge (i.e., tolerance tests) with a combination of fat, carbohydrates and protein has been successfully used to disturb homeostasis [33,34]. Drawing conclusions on the metabolic health status of an individual based on single biomarkers is challenging as it may provide an incomplete picture; thus, an aggregate marker could be calculated by integrating multiple metabolic markers into a composite score [37,38].

The objective of the current study was to determine the impact of an integrated personalized systems nutrition (PSN) program in a workforce. The PSN program included personalized dietary advice based on individual phenotype (challenge test response), genotype, and anthropometric data in combination with participant-generated data on diet, physical activity, goals, and preferences. The personalized dietary advice was provided via recipes and macro- and micronutrient recommendations, but also in the form of ready-made meals (breakfast and lunches). The PSN program included behavior guidance through individual coaching and motivational interviewing and behavior change promotion through goal setting, positive feedback and self-monitoring. The effect of this 10-week PSN program on lifestyle behavior change, including dietary intake, activity, and sleep, was evaluated. Furthermore, the effect of this program on health outcomes, including individual markers and an aggregate score for metabolic health status (i.e., health space model) was also evaluated [39,40].

## 2. Materials and Methods

### 2.1. Study Design

**Figure 1:**
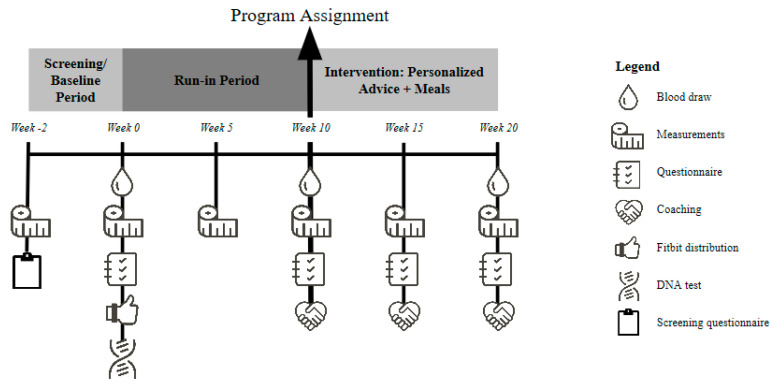
Study design overview. The screening visit, run-in period and 10-week intervention (personalized advice and meals; phase 1) of a single-arm, multi-phase study. Screening consisted of anthropometric measurements and a screening questionnaire. After screening, participants had mid- and end-point visits/contacts during the run-in and intervention period. Participants completed an at-home challenge test and sample collection (weeks 0, 10 and 20; including DNA at week 0 only), anthropometric and body composition assessments (all weeks), electronic questionnaires (all weeks except 5), coaching (weeks 10, 15 and 20), and were distributed an activity tracker (Fitbit; week 0).

This was a single-arm, multi-phase, open-label exploratory trial that consisted of four 10-week periods preceded by a screening session (week −2). Each of the periods had a mid- and end-point visit: (i) baseline (week 0) and run-in (week 0–10), (ii) intervention phase 1: personalized coaching/advice and meals (week 10 to 20), (iii) intervention phase 2: personalized coaching/advice (week 20 to 30), (iv) follow-up (week 30 to 40, endpoint visit only). The focus of this manuscript is on the methods and main results from data collected at baseline, during run-in and in phase 1 of the study, i.e., through week 20 (Figure 1). Due to a higher-than-expected dropout from phase 2, these data were excluded from the analysis. The study was conducted in accordance with Good Clinical Practice Guidelines, the Declaration of Helsinki [41], and the United States 21 Code of Federal Regulations. An institutional review board (Hummingbird IRB, Needham, MA, USA) approved the protocol before initiation of the study, and participants provided written informed consent before implementation of any study-specific procedures. This study was registered at clinicaltrials.gov (accessed on 18 March 2021) as NCT03424395, which includes details of the study design and outcomes assessed.

### 2.2. Participants

Generally healthy men and women, 30 to 65 years of age, with body mass index (BMI) 18.5 to 39.9 kg/m^2^ were recruited from a workforce (Campbell Soup Company, Camden, NJ, USA). Eligibility was assessed via a screening questionnaire (week −2). Eligible participants were those who met the inclusion criteria, were willing to follow all study procedures and who had access to an internet-ready device and a functioning personal email address. Participants were deemed ineligible based on the following exclusion criteria: a history or presence of diagnosed conditions that could interfere with study outcomes, uncontrolled hypertension, a current or recent history of nicotine or heavy alcohol use (>14 drinks per week), current or recent use of lipid altering medications, allergy or sensitivity to the study foods provided or very specific dietary habits (e.g., vegan, very low carbohydrate), or a recent history of body weight change >10%. A complete description of all inclusion and exclusion criteria can be found on clinicaltrials.gov (accessed on 18 March 2021) (NCT03424395).

### 2.3. Study Procedures Overview

At each visit, anthropometrics (height (first visit only), weight, fat mass, waist and hip circumference) [42] and blood pressure were assessed according to standard operating procedures by the study coordinator.

Validated questionnaires were administered electronically (REDCap Cloud, version 1.3, Encinitas, CA, USA) in weeks 0 (baseline), 10, 15 and 20. Additionally, participants were provided with an activity tracker at baseline (Charge 2, Fitbit, San Francisco, CA, USA) and were asked to wear the device for the remainder of the study.

In weeks 0, 10 and 20, participants were provided with an at-home kit including all necessities for challenge testing and dried blood spot (DBS) and DNA (week 0 only) sample collections. Prior to testing at baseline, participants were provided with private access to a digital platform which included video instructions and an on-boarding form for logging age, body weight and height, hypertension status (yes or no), waist circumference and physical activity history. Data from this form was used along with clinical and DNA results to generate personalized recommendations.

Personalized recommendations were provided to participants through a digital platform on week 10. Additionally, breakfast and lunch meals were provided and tailored to their macronutrient recommendations, five days a week for nine weeks, starting at week 10. Meal diaries were collected weekly to assess compliance. Video and phone coaching sessions were scheduled with participants in weeks 10, 15 and 20. Adverse events were evaluated at the beginning of each visit, except at screening.

### 2.4. At-Home Sample Collection and Challenge Test

Participants were instructed to avoid vigorous physical activity and fast for 10 to 14 h (water only) prior to completing the at-home kit. Sample collection began with buccal cell collection by cheek swab (week 0 only for DNA isolation) followed by fasting capillary blood (0 min). Challenge beverages were then consumed within a 5-min time period and capillary blood collected at 30- and 120-min post-beverage consumption. All capillary blood was collected on DBS cards (Advance DX100, Advance DX, Inc., Chicago, IL, USA). Participants placed their DBS cards and cheek swabs in packets and brought them to the study office where they were logged and shipped for analysis (Aegis Sciences Corporation Nashville, TN, USA).

A nutrient dense mixed-meal beverage was used for challenge testing, which has been previously shown to effectively perturb metabolic homeostasis [43]. The challenge beverage (414 mL; Jasper Products, Joplin, MO, USA) consisted of 60.1% (*w*/*w*) water, 13.6% (*w*/*w*) palm oil, 18.9% (*w*/*w*) dextrose, 5.3% (*w*/*w*) milk protein isolate, and <1.5% each of vanilla, cassia flavor, trisodium citrate, canola lecithin, and gellan gum. This resulted in a beverage of 3950 kJ/950 kcal with a macronutrient composition of ~64 g fat, 22 g protein and 88 g carbohydrate. 

### 2.5. Personalized Systems Nutrition Program

Decision trees and algorithms were used to generate personalized dietary recommendations using individual on-boarding data (including self-reported body weight, waist circumference and blood pressure), clinical measures (including measures before and after a mixed-meal challenge test) and single nucleotide polymorphism (SNP) variants (Table 1) according to the proposed guidelines to evaluate scientific validity and evidence for genotype based dietary advice [44]. SNPs indicated in bold (Table 1) drove personalized dietary recommendations if the risk-variants of these SNPs coincided with an unhealthy phenotype. All other SNPs were only used to help provide additional context and supporting recommendations (Supplementary Table S1). Individuals were grouped into seven possible personalized diet types (PDTs) that differed in terms of phenotypic flexibility (A = highest possible flexibility to G = lowest flexibility). Personalized advice for these PDTs differed in terms of macronutrient profiles that met the USDA Dietary Guidelines for Americans [1] and/or Acceptable Macronutrient Distribution Ranges [45] (Table 2). PDTs were determined using onboarding data and clinical measures. SNPs did not independently determine diet type. If a risk variant for FTO rs9939609 coincided with a high waist circumference, this led to a high protein and low fat and carb PDT [46,47,48]. Energy intake advice was determined based on total energy expenditure. The basal metabolic rate was calculated using the Mifflin St. Jeor equation [49], which was then multiplied by the daily physical activity level (PAL) score. The total daily PAL was calculated as the sum of the daily pattern PAL score based on a categorization by Hall et al. and PAL scores for sport and leisure activities [50,51]. Micronutrient recommendations were determined using onboarding, anthropometric and clinical measures and further supported by SNP data if physiological pathways were known. For instance, it has been shown that in hypertensive people with the MTHFR rs1801133 risk variant riboflavin supplementation may contribute to blood pressure lowering [52,53]. Finally, SNP-based narratives were provided for a few food-related sensitivities, physical activity and vitamin D, which describe the linkages between the SNP and certain recommendations or health outcomes, but do not imply causality (Table S1).

In addition to the seven PDTs that guide macronutrient recommendations, the algorithms generated micronutrient and calorie recommendations. Micronutrient recommendations were personalized by age and gender (per U.S. RDAs), dietary intake, clinical measures and SNP data [54,55]. 

Finally, participants were provided with personalized recipes and meals according to their macro- and micronutrient recommendations [56]. Participants had access to a digital platform which included their personalized recommendations, test results and narratives explaining the participant’s clinical, genotypic and anthropometric data. Additional information on the decision trees and algorithms that were used can be provided upon request. 

Coaching by a registered dietitian nutritionist (RDN) occurred three times for phase 1 (week 10 to 20) (Figure 1). RDNs were trained on coaching techniques as well as the algorithms behind the PSN program prior to the start of phase 1. Coaching sessions were provided by two RDNs who jointly developed the format of the sessions based on behavior science and held, at minimum, bi-weekly conference calls to review content for participants’ sessions. During the first coaching session (week 10), which was a video conference, the RDN explained their clinical results to each participant and how they were linked to their personalized dietary recommendations. Additionally, readiness to change and self-efficacy were explored [57,58]. After the first coaching session, participants were instructed to set personal goals for at least one goal area (modifying eating behavior, exercise, sleep, general balance and mindfulness) using SMART (specific, measurable, attainable, realistic, time-based) goal setting criteria [59]. To provide participants with a foundation for change, they were shown graphics and data during their session comparing their present reported dietary macro- and micronutrient intakes to their personalized program recommendations. Participants were provided the graphics and data electronically after the session for reference. Additionally, they were provided with food recommendations and considerations to help them achieve their program plan. During the second coaching session (week 11), delivered by phone, personalized behavior change SMART goals using the SMART criteria were reviewed and finalized. During the third coaching session (week 15) participants could touch base on goals or other issues related to their personalized program. Participants where shown their most recent food intake data compared to the personalized recommendations. Individual’s chosen goals were reviewed to assess their progress and adjust behavior change as needed. This was followed by an electronically delivered report recapping the session. Coaches used motivational interviewing strategies and techniques to facilitate behavior change at all sessions and contacts [57,58]. In addition to coaching, throughout phase 1, participants received information on how to follow their personalized diet via email and on their digital platform. This information included guidance on meals and snacks, eating out, and recipes for their PDT.

### 2.6. Study Meals and Compliance

Participants received tailored breakfast and lunch five days a week for nine weeks, beginning at week 10. All meals were prepared on-site (Sodexo Food Services, Gaithersburg, MD, USA) according to macronutrient distributions per assigned PDT (Table 1). When possible, food preferences were accommodated. Participants were provided with meal diaries weekly and asked to record how much of each meal they consumed. Responses were scored as follows: ‘I did not eat’ (0), ‘≤50%’ (0.5), or’ ≥50%’ (1) and compliance was calculated as the percentage of meals consumed based on the number of meals provided.

### 2.7. Dietary Intake

Participants recorded all food and beverage intake consumed over three days (two weekdays and one weekend day) using a standard dietary record methodology prior to all visits except for the screening and week 5 visits [60]. At randomization, participants were instructed on how to collect dietary recalls, and shown household measuring cups, spoons and a ruler, and instructed on how to obtain portion sizes on labels. The records were reviewed by an RDN who followed up by email if clarification was required. Records were analyzed using Food Processor Nutrition Analysis Software (version 11.6, ESHA, Salem, OR, USA) and nutrient intake and calories were averaged over the three days and used for statistical analyses.

### 2.8. Anthropometrics and Vitals

At each visit, anthropometrics (height (first visit only), weight, fat mass, waist and hip circumference) and blood pressure were assessed. Duplicate measures for body weight and fat were obtained using the BC-554 IRONMAN^®^ Body Composition Monitor (Tanita, Arlington Heights, IL, USA) according to standard methodology as provided by the Tanita BC-554 scale. The Tanita BC-554 model has single frequency bioelectrical impedance analysis technology to assess changes in body fat and fat mass over time. The same Tanita scale was used for all participants throughout the entire duration of the study. Our protocol aimed to control for the effects of hydration state, body temperature, and time of day on measurements by educating participants on hydration status and conducting clinic visits at similar times. Waist and hip circumference were performed by the same study coordinator following the WHO standards [42]. Triplicate measures for blood pressure (Home™ 1500 Series Upper Arm Blood Pressure Monitor, Welch Allyn, Chicago, IL, USA) were taken according to standard operating procedures and the last two measurements were averaged.

### 2.9. Wellbeing and Lifestyle

Dietary behavior was assessed using the validated 34-item Adult Eating Behaviors Questionnaire (AEBQ) [61] which was administered electronically prior to visits at weeks 0 and 20. Quality of life (QOL) was assessed using the validated 26-item WHOQOL-BREF questionnaire [62], which was administered electronically at week 0, 10 and 20.

Participants wore an activity tracker (Charge 2, Fitbit, San Francisco, CA, USA) from week 10 to 20 for assessment of daily activity (heart rate, number of steps) and sleep hours. Data was collected and stored using Fitabase (Small Steps Labs, San Diego, CA, USA) prior to analysis.

### 2.10. Laboratory Analyses

All laboratory analyses were performed by Aegis Sciences Corporation (Nashville, TN, USA). DNA was isolated from buccal samples and analyzed for quantity and quality using an RNaseP assay. A panel of SNPs, associated with dietary intake-related phenotypes, was investigated using qPCR on the TaqMan/Life Tech Platform™ (Thermo Fisher Scientific, Waltham, MA, USA).

A 0.49-inch sample was punched from the serum eluded on the DBS cards. These cards are designed to separate the serum from cellular components of the whole blood and thus are subject to hematocrit bias/effect [63]. From this sample, serum glucose, triglycerides, total cholesterol and HDL cholesterol were analyzed using enzymatic colorimetric tests on an Olympus 5400 (Olympus Corporation, Tokyo, Japan). LDL cholesterol was calculated using the Friedewald Equation [64]. A standard sandwich ELISA kit was used to assess C-peptide (Mercodia, Upsala, Sweden) using a Freedom EVO 150 platform fitted with a Columbus microplate washer and Sunrise microplate reader (Tecan, Mannedorf, Switzerland). All test results were normalized to total microprotein concentrations. Each normalized result was projected to a serum concentration using algorithms generated by Aegis Sciences Corporation.

### 2.11. Calculation of Insulin Sensitivity Indices

The glucose and C-peptide values derived from the challenge tests at all timepoints were used to calculate the following indices: simple Matsuda index and Homeostatic Model Assessment for Insulin Resistance (HOMA-IR) as measures of whole body insulin resistance [65,66].

### 2.12. Statistical Analysis and Data Management

An evaluable sample of 100 participants was expected to provide 80% power assuming an alpha = 0.05, two-sided, and an effect size of 0.3 for health space score based on a previous nutritional intervention study [67].

**Figure 2:**
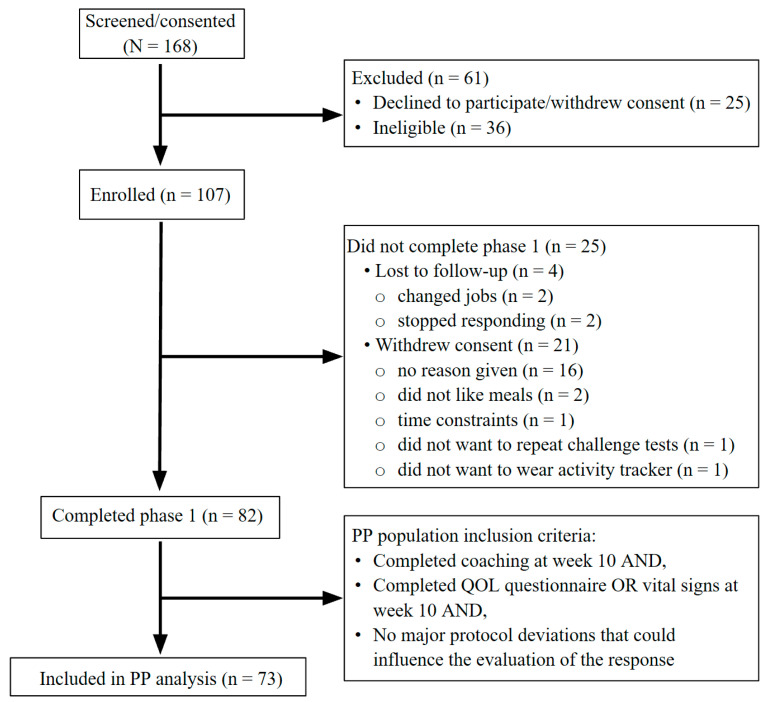
Study flow diagram. A total of 168 participants were screened/consented and healthy men and women were enrolled in the study (*n* = 107). A total of 82 participants completed phase 1 (personalized advice + meals intervention period; through week 20). Of the 25 participants that did not complete phase 1, four were lost to follow-up and 21 withdrew from the study. Data from 73 participants were included in the PP analysis. Abbreviations: PP, per protocol; QOL, quality of life.

A sample of 107 participants was enrolled to account for attrition and/or non-compliance (Figure 2). Tests of significance were performed at α = 0.01 for questionnaires and Fitbit data and at α = 0.05 for all remaining tests. The primary outcome variable was the health space score. All remaining outcomes were secondary. The analysis was completed on a per protocol (PP) population, which was defined as follows: completing coaching session at week 10, and either completing a key questionnaire (WHOQOL-BREF) or vital signs and anthropometrics, with no major protocol deviations.

#### 2.12.1. Health Space Model

**Figure 3:**
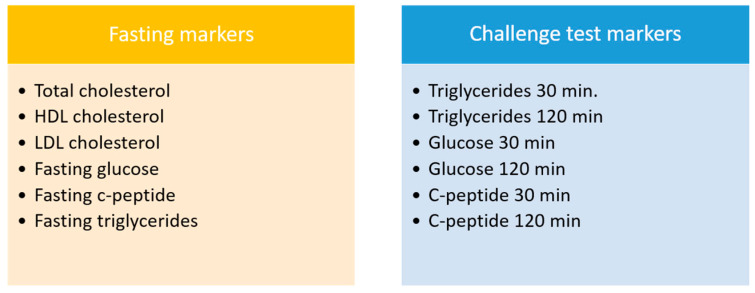
Data used in the health space model. Abbreviations: HDL, high-density lipoprotein; LDL, low-density lipoprotein; TG, triglycerides. Postprandial markers were measured at 30 and 120 min after challenge beverage consumption.

The health space analysis is a multivariate supervised dimension reduction method that serves to summarize multiple variables into a single biologically meaningful score. Ridge regression was the applied method for the creation of health space models [68]. The model is a trained classifier that discriminates between two predefined reference groups [40]. During the training procedure, 10-fold cross validation was used to find the optimal shrinkage parameters for the model as well as to determine model quality using the misclassification error. The data for each of the variables in the input dataset was centered on the mean and scaled by the standard deviation (Figure 3).

The reference groups were taken from previous research which aimed to create a health space representative of the normal range of health, using the phenotypic flexibility concept [40]. On the low end of the spectrum is the young and lean reference group (20 to 29 years of age, normal body fat percentage, which was <20% in male and <30% in female) while the high end of the spectrum is represented by the older group with a higher body fat percentage (60 to 70 years of age, body fat percentage ranging from normal to high, which was >20% in male and >30% in female) [40]. The number that is produced by providing this model with data from the study participants is termed the ‘health space score’.

In summary, the health space score presented here aligns with the range of metabolic states within a normal healthy population. A higher score represents reduced phenotypic flexibility and a higher degree of similarity with older people with higher adiposity, while a low score suggests a greater degree of resilience and a higher degree of similarity with a young lean group.

#### 2.12.2. Wellbeing Questionnaires and Activity/Sleep Data Analysis

For the WHOQOL-BREF [62] and the AEBQ [61] the (sub)scales were calculated according to the official guidelines.

The Fitbit data provided information on the number of steps taken, resting heart rate and the hours of sleep. For the number of steps taken, the average steps per day over the phase preceding the measurement point was used. For resting heart rate and hours of sleep a similar approach was used.

The questionnaire and Fitbit data were evaluated using a linear mixed effect model where the intercept is dependent on the individual; this is akin to a repeated measurement model. Due to the nature of the data, PDT, gender and timepoint were used as explanatory variables. When multiple time points were evaluated, time point was included as a covariate. The assumptions of linearity, normality and homoscedasticity were checked for each significant model. A 0.01 level was used to justify a claim of a statistically significant effect. Statistical analyses were completed using R software (version 3.5.1; The R Foundation, Vienna, Austria).

#### 2.12.3. Linear Mixed Model Univariate Analysis

All remaining variables were assessed by univariate analysis using linear mixed models. For the univariate analysis, linear mixed models were used. All variables were LOG transformed before statistical analysis. A mixed model was used for statistical analysis. In this model, the focus was on visit and PDT including its interaction. Age, gender and cohort were three covariates in the model. In this model age, gender, cohort, visit, PDT and PDT × visit were fixed factors. The participants within a cohort represented the random factor. If significant effects were observed, post-hoc tests were applied. To correct for multiple testing, a Tukey–Kramer multiple comparison correction was applied on the *p*-values of the post-hoc tests. Assumptions of normality and homoscedasticity were investigated by graphical representations on residuals produced by statistical models. If the model residual of any data point was larger than 3 × RMSE (root mean squared error) for a certain variable, the data point was considered as a statistical outlier for this variable and removed from the particular data set before creating a new model. For all statistical tests using the linear mixed model, a 0.05 level was used to justify a claim of a statistically significant effect. The tests conducted were two-sided. This analysis approach was used for all data except for the psychological questionnaire and Fitbit data. Statistical analyses were conducted using SAS version 9.4 (SAS Institute Inc., Cary, NC, USA).

## 3. Results

### 3.1. Study Logistics and Baseline Characteristics

Between October 2017 and February 2018, 168 individuals were recruited and assessed for eligibility (Figure 2). Initially, 107 participants were enrolled in the study. A total of 82 participants completed the phase 1 intervention (week 10 to 20). The per protocol (PP) population included a total of 73 participants (Table 3).

Participants mainly selected modifying eating behavior as their primary goal (~56%) during the coaching sessions, followed by exercise (~33%) and mindfulness (~11%). None of the participants selected sleep or general balance as a primary goal.

Within the PP population, two PDTs were mainly represented, which were group A (*n* = 48) and group G (*n* = 22); only three participants were classified into one of the other PDT categories (group B, E and F). For this reason, we restricted the discussion to diet type groups A and G only. As a result of the automated PSN algorithms, which assigned one of seven PDTs based on metabolic health status at baseline, groups A and G represent the most and least phenotypically flexible groups, respectively. Group G had a higher degree of adiposity and a higher age as compared to group A (Table 3). No differences in dietary patterns were observed at baseline between the two PDTs. Both groups were 82% compliant with personalized meal intake.

### 3.2. Run-in Period Effects (Qualitative Control)

The run-in period (week 0 to 10), which was the same duration as the intervention period, provides an indication of behavior and health effects of being included in a clinical study (without being provided with the personalized nutrition program) and served as a qualitative control. In terms of dietary intake, total fat intake increased (+7.5 g; *p* = 0.037) from week 0 to 10 (Supplementary Table S2). This may be attributed to a higher intake of monounsaturated fatty acids (MUFA) (+4.3 g; *p* = 0.003) and polyunsaturated fatty acids (PUFA) (+2.5 g; *p* = 0.003). For group G only, there was a decrease in beta-carotene intake (−1031 mcg; *p* = 0.007) (results not shown). Additionally, some small but unfavorable health differences were found. Fasting and 2 h C-peptide (+0.11 nmol/L *p* = 0.009 resp. +0.25 nmol/L; *p* = 0.003) as well as 2 h glucose levels (+0.31 mmol/L; *p* = 0.042) were elevated after the run-in period. Consequently, HOMA-IR (+26.6%; *p* = 0.001) increased, and Matsuda index decreased (−15%; *p* = 0.0004), indicating an increased state of whole-body insulin resistance. 

### 3.3. Intervention Effects

#### 3.3.1. Dietary Intake

Many dietary intake changes were observed. For the PP population, intake of calories (−256.2 kcal; *p* < 0.0001), carbohydrates (−22.1 g; *p* = 0.0039), sugar (−13.0 g; *p* < 0.0001), total fat (−17.3 g; *p* < 0.0001), saturated fat (SFA) (−5.9 g; *p* = 0.0003) and PUFA (−2.5 g; *p* = 0.0065) were reduced during the intervention (week 10 to 20) (Table 4 and Table S2).

Energy intake from fat was significantly reduced and there was a small but significant increase in energy intake from fiber. When looking at differences between the PDT, percent calories from protein significantly increased in group G and not in group A, reflecting compliance with personalized dietary advice for these groups (Table 2 and Table 4).

For micronutrient intake, significant increases were seen during the intervention period for vitamin C (+33.6 mg; *p* = 0.0002), magnesium (+47.7 mg; *p* = 0.0029) and potassium (+327.4 mg; *p* = 0.0328) in the PP population. Finally, sodium levels were significantly reduced (−546.0 mg; *p* = 0.0007). When looking at subgroups, a significant increase in beta-carotene intake was seen in group G, but not group A.

#### 3.3.2. Wellbeing and Lifestyle

For the PP population, the total steps per day increased, and the resting heart rate decreased during the intervention (Table 4 and Table S2).

In terms of eating behavior, the only change observed was a higher satiety response after a meal in the PP population.

A significant positive correlation between the WHOQOL physical scale score at week 10 and the number of steps taken during intervention (correlation = 0.447; *p* < 0.001) was observed. Additionally, a positive correlation between the change in steps over time (change in steps from run-in period as compared to intervention period) and the WHOQOL health scale score at week 10 was observed (correlation = 0.39; *p*< 0.01).

#### 3.3.3. Anthropometrics and Vitals

**Figure 4:**
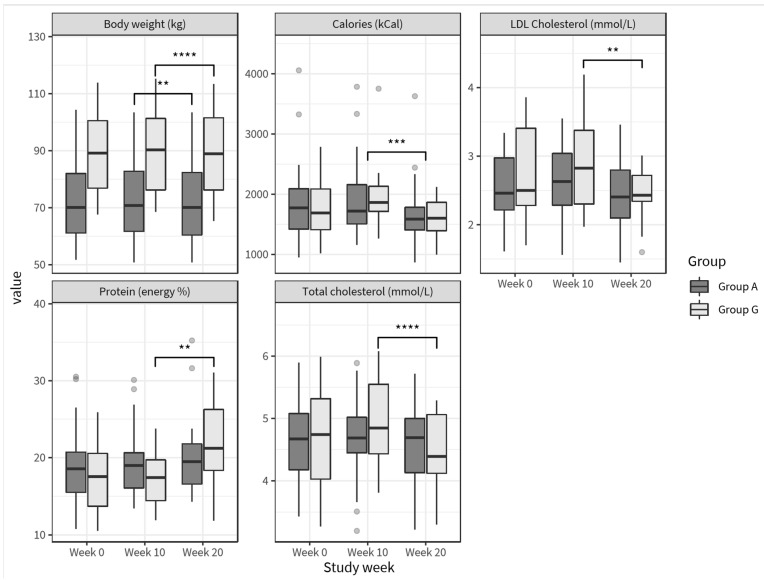
Boxplots of body weight (kg), protein intake (energy %), calorie intake (kcal), LDL cholesterol (mmol/L) and total cholesterol (mmol/L), grouped according to personalized diet type; dark grey box plots represent group A (*n* = 48) and light grey box plots represent group G (*n* = 22). Subgroup specific statistically significant differences are noted (** *p* < 0.01; *** *p* < 0.001; **** *p* < 0.0001) over time, except for calories where a statistical difference for the PP population is indicated.

After the intervention period (week 10 to 20), during which participants received personalized dietary recommendations, coaching and personalized meals, several health improvements were observed for the PP population. While this was not designed to be a weight loss trial, BMI (−0.6 kg/m^2^; *p* < 0.0001), body fat (−1.2%; *p* = 0.0192) and hip circumference (−5.8 cm; *p* < 0.0001) showed a significant decrease from week 10 to 20 for the PP population (Table S2), and body weight significantly decreased after the intervention in both group A and group G (Table 5, Figure 4).

#### 3.3.4. Clinical Chemistry

For group G, significant reductions were observed in LDL cholesterol and total cholesterol during the intervention (week 10 to 20) (Table 5, Figure 4). 

#### 3.3.5. Health Space

**Figure 5:**
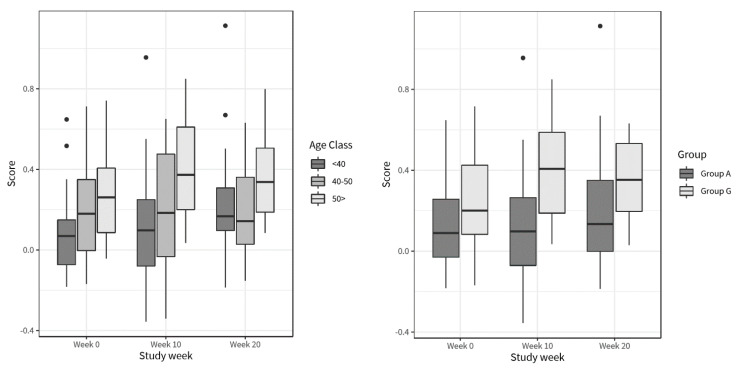
Boxplots of health space scores for baseline (week 0, *n* = 73), end of run-in (week 10, *n* = 63) and end of intervention (week 20, *n* = 49), grouped according to age (left) and personalized diet type (right). A lower score on the health space is considered healthier.

A health space score was calculated where complete datasets were available (*n* = 63 at week 10 and *n* = 49 at week 20). Analysis of the health space showed that both biological age and BMI were positively correlated with health space scores (Figure 5). A higher health space score reflects lower metabolic health status. No differences in health space scores were observed between group A and group G (*p* = 0.474). Furthermore, no changes in health space scores due to the intervention were found for either the PP population (*p* = 0.380) or subgroups (*p* = 0.113).

## 4. Discussion

This study shows that a PSN program in a workforce improves dietary habits and physical activity and reduces body weight, BMI and other health-related outcomes. These changes were most pronounced in group G, the subgroup with a compromised phenotypic flexibility at baseline.

While this study was not designed to promote weight loss, the PSN program resulted in an overall reduction in caloric intake, and an improvement in diet quality, as reflected by a decreased intake of total and saturated fat, sugar and sodium. Additionally, a decrease in absolute intake of PUFA was seen, but as there were no changes in energy percentage from PUFA, this is likely caused by the reduction in caloric intake. Overall, the reduction in total and saturated fat intake, sugar and sodium during the intervention period reflects an improved diet quality. This is further underscored by improvements in micronutrient intake, in terms of increased vitamin C, magnesium, potassium and beta-carotene intake, suggesting a higher intake of fruits and/or vegetables [69,70,71]. An increase in fruit and vegetable intake is in line with the personalized meals, recipes and suggestions for both subgroups. Overall, the nutrient data suggests that after the personalized intervention, participants showed eating habits that are more aligned with population-based dietary recommendations. When looking at subgroups, the PSN program not only improved adherence with population-based dietary guidelines, but also better alignment with personal needs was achieved. For group G, different positive effects were seen with respect to dietary intake, consistent with their respective dietary recommendations. Protein (as a percentage of total energy) and beta-carotene intake increased during the intervention period in group G, but not in group A. The increase in protein intake can be directly related to the personalized advice of group G, as they were recommended to consume a diet high in protein and low in carbohydrates and fat. The increase in beta-carotene intake could be the result of a higher intake of fruits and vegetables. It should be noted that the personalized ready-made meals for group G were higher in protein and beta-carotene compared to the meals for group A. These results indicate that personalized nutrition programs may be effective in motivating people to consume a diet that meets individual needs while moving them closer to public health recommendations overall. As such, personalized nutrition programs seem to have added value as compared to general guidelines or one-size-fits-all approaches. Therefore, personalized offerings could be an interesting strategy in improving adherence with general dietary guidelines, by using general dietary guidelines as the basis for personalized nutrition and further finetuning these recommendations to individual needs and preferences.

Besides improvements in dietary intake behavior, the intervention resulted in increased physical activity in the PP population. Additionally, a small decrease in resting heart rate was apparent, which could indicate improved physical fitness [72,73,74]. However, it is unclear whether a small reduction in 1.3 bpm resting heart rate is clinically meaningful. The improvement in physical activity is an interesting finding, as the intervention consisted of dietary advice only and participants did not receive any recommendations on their physical exercise. This increase in physical activity thus seems to be a beneficial side-effect of being involved in the PSN program.

The improvements in dietary intake and physical activity are supported by improvements in health parameters during the intervention, including body fat, BMI, body weight, hip circumference and total and LDL cholesterol. The reduction in body fat, BMI, body weight and hip circumference are relatively small and may not be clinically meaningful. A 3 to 5% of weight loss can be considered clinically meaningful, while in our study weight loss was ~1% [75]. However, this was achieved with a normal diet that did not focus on weight loss. These declines may become clinically meaningful if they persist with a continued healthy eating pattern. The reduction in BMI, body fat and hip circumference in the PP population during the intervention period can be explained by the reduced calorie intake and increased step count, suggesting a negative overall energy balance [76,77,78].

When looking at differences between the two PDTs, the degree of weight loss was more consistent for group G (*p* < 0.0001) as compared to group A (*p* < 0.01). At baseline, group G already had a significantly higher BMI as compared to group A, potentially leaving more room for improvement. However, body weight was not significantly different between groups. One could argue that the more consistent weight reduction in group G could be a result of higher protein intake, which has been shown to aide in weight loss [79,80,81,82], possibly as a result of improved satiety, appetite and diet-induced thermogenesis [83,84]. This was also reflected in the increased satiety response during the intervention, although this change was seen for the PP population and was not specific for group G.

The decreased total and LDL cholesterol for group G may be partly explained by the reduced total and saturated fat intake during the intervention, as it has previously been shown that a higher saturated fat intake is correlated to higher total and LDL cholesterol levels [85,86,87]. However, the decrease in saturated fat intake during the intervention was similar for group A and G. Additionally, this decrease in total and LDL cholesterol in group G cannot be ascribed to baseline between-group differences and is likely a result of the PSN program. It has been proposed by the International Society of Nutrigenetics/Nutrigenomics (ISNN) that personalized advice should be more effective in preventing chronic disease than population-based dietary guidelines [88]. This study indeed suggests that personalized advice may be important to achieve desired health and functional outcomes. Additionally, the differential effects between subgroups indicate the added value of personalization. This suggests that personalized nutrition may enable changes in dietary intakes that have not occurred through public health recommendations. Previous research comparing DNA-based dietary guidelines with population-based dietary guidelines indeed showed greater changes in the intake of specific dietary components in the personalized group [89,90]. This could be explained by the fact that dietary guidelines only distinguish recommendations based on gender and age, whilst personalized nutrition can use more specific and detailed personal information in generating relevant dietary advice. While more research is needed to see if these changes can be sustained over time, the results suggest that personalized approaches to health may be more effective than general guidelines and mass media campaigns for achieving dietary goals. In addition to providing a means to improve health, it also provides a means to work more closely with regulators. Moving from population-based programs to personalized recommendations and claims is new to many regulators. Personalized approaches can align to and support adherence to population-based guidance, which may help personalized programs gain greater acceptance [13,91].

Despite the differences in individual measures of health, no overall health effect could be observed using the health space score. Furthermore, there were no significant differences in health space scores between group A and G. This may be ascribed to the small number of participants with sufficient data for health space analysis (*n* = 46), whilst the power calculation indicated that data for 100 participants were required to detect a significant change. Additionally, the intervention period was only 10 weeks, which is relatively short to achieve significant changes in the total set of biomarkers. As there were substantial improvements in dietary intake and markers of health status, it could be expected that changes in health space may have been observed with a longer intervention. Furthermore, the largest subgroup in the study consisted of group A (*n* = 48), representing subjects who were most phenotypically flexible. For this PDT, there may have been less opportunity for health improvement based on the markers used, as opposed to the smaller group G (*n* = 22), which forms the least flexible PDT. Despite this limitation, we were still able to show an overall improvement in dietary behavior in our study as well as on single health outcomes.

Our findings are consistent with previous studies on personalized nutrition programs, which also show benefits of personalized advice as compared to a control group [9,92]. Previous reports have suggested that it is unclear whether personalization based on phenotype or genotype has additional value as compared to only using dietary intake for personalization [93,94]. A recent systematic review on the effect of incorporating genetic testing results into nutrition counseling on dietary intake concludes that disclosure of genetic information in carriers of high-risk gene variants may produce benefits, but results should be interpreted with caution due to the limited number of studies and large heterogeneity [95]. In the present study, phenotype and genotype, and not dietary intake data, were used to create PDTs. However, the included SNPs only played a minor role in the personalized advice, and thus probably had a limited effect on the study results. Our results mainly demonstrate that biological markers can be effectively used for personalization of advice leading to improvements in diet quality and health status. For example, we found improved total and LDL cholesterol and more consistent weight loss in the subgroup with a reduced health status. This beneficial effect might not have occurred if the personalized advice would have been based on dietary intake information only. A recent consensus report from the Academy of Nutrition and Dietetics also states that personalized nutrition requires a holistic approach that reflects lifestyle, preferences, health status and other domains of nutrition care [96,97].

It has been recognized previously that not only the information used for personalization of advice is of importance, but personal goals, barriers and preferences are also essential in the adoption of lifestyle changes [98]. The incorporation of these factors in our study, and thereby taking a holistic approach to personalized nutrition, may partially explain the intervention success. Surprisingly, an individual’s perceived health and quality of life also seems to influence intervention success. In this study, a higher self-reported physical health score at baseline was associated with a higher number of steps after the intervention. Additionally, a higher perceived health was associated with a larger change in steps during the intervention period. This suggests an association between steps or physical activity and health satisfaction. It has been previously reported that a lower perceived physical and psychological health can form barriers for lifestyle behavior change [99,100]. In other words, people with a lower self-reported quality of life may experience more barriers for lifestyle behavior change, which may result in a lower effectiveness of lifestyle interventions. It has been shown that behavioral treatment strategies, including goal setting and motivational interviewing, improve adherence to lifestyle intervention programs [16,101]. The incorporation of such strategies in our personalized nutrition program may explain the high (82%) compliance rates with the personalized meals in this study. The results underscore the importance of providing both personalized dietary recommendations based on an individual’s biological data as well as tailoring behavior advice to achieve better compliance.

Investigating the sustainability of intervention adherence and the beneficial effects of our PSN program over time would require long-term follow up of participants. However, it has been shown previously that challenge testing is a highly sensitive approach in detecting subtle changes in health [35,37], which could allow for fine-tuning the personalized advice to changes in health status over time. In the future, it could be interesting to consider an *n*-of-1 approach, which focuses on changes over time within an individual and could therefore help identifying differences in effectiveness of personalized programs between subjects and subgroups on a more detailed level.

### 4.1. Limitations

There were some limitations to this study that should be considered. First, by design, the distribution of participants over the subgroups could not be influenced, as the automated PSN algorithms assigned participants into one of seven PDTs after enrollment. Unfortunately, this resulted in an unequal distribution, with only two out of seven PDTs frequently occurring.

Second, self-measurements were used for generating the personalized dietary advice. During the onboarding process, participants self-measured their body weight, height, and waist circumference and reported on hypertension status (yes or no). When using waist circumference and blood pressure data as assessed by the study team during the baseline visit instead of the self-reported data for assigning the PDT, 22 participants in group A should have been classified as group E. Most misclassifications occurred because hypertension was not reported by hypertensive participants, even though some of these participants were aware of their hypertension. If objective measurements would have been used, and participants would have been categorized accordingly; being confronted with their compromised health status may have motivated them to change their behavior to a larger extent. Accurate classification of the 22 participants to group E may therefore have resulted in larger differences between the subgroups. This underscores that caution should be exercised when using self-reported data for personalized services. Misreporting may in general have consequences for the success of personalized nutrition programs, if these programs rely on self-reported data. In addition, an inherent limitation to the use of the bioelectrical impedance is hydration status, which may ultimately result in the misestimation of fat and fat-free body mass. This misestimation may be more prevalent in obese individuals due to differences in body water, relative to normal weight individuals [102]. We did attempt to minimize these limitations in our design where participants served as their own control, by guiding participants on the importance of consistent hydration, in the use of consistent equipment and similar timing of visits. Finally, although well-described and standardized, the procedure used for collecting dietary intake data was not internally validated. Another limitation in this study was the lack of a control arm in this study. As this study was conducted in a workforce setting, a naïve control was not possible due to the inability to blind participants to the intervention. However, a 10-week run-in period was part of this study, which provides an indication of behavior changes and health effects of being included in a clinical study and could therefore be used as a qualitative control. During this run-in period, insulin resistance parameters increased, suggesting a reduced health state during the run-in period. The health improvements during the intervention period can therefore be ascribed to the PSN program and are not merely the result of being involved in a clinical trial. In a follow-up study, the effects of the PSN program should be compared to a control group receiving general advice.

Last, the intervention took place in a workforce. Previous studies have shown that sorting beneficial health effects in a workforce is challenging, which includes issues such as fit with organizational values, work climate, (perceived) management support, low participation rates and restructuring [103,104,105,106]. This workforce setting may also explain the drop-out rate in this study, as other workplace prevention programs show high attrition rates of 30 to 50%, whilst more intensive participation in workforce programs has been correlated with a greater reduction in health risks [107,108,109].

### 4.2. Strengths

First, despite the challenges related to performing a study in a workforce setting, this setting is also a strength of this study. As the workforce setting is a potential implementation area for personalized nutrition programs, performing a study in such a setting provides a good indication of its effectiveness in real life. Even in this real-life situation, beneficial effects of a personalized nutrition program were found.

Second, the onboarding for this personalized nutrition program was also designed such that it was completely do-it-yourself and thus could be performed in an at-work or at-home setting. For blood collection, DBS cards were used and required only a few blood drops that were easily collected by finger pricks. Blood spot collection was completed unsupervised. The type of card used allowed for multiple samples to be collected from each card to help correct for under-sampling on a given card. Despite some of the limitations discussed above, this report demonstrates that a do-it-yourself personalized nutrition program can improve diet and markers of health status.

Third, the personalized nutrition program combined an online platform with feedback, advice, and contact with an RDN, which augmented the experience for participants. Additionally, previous research has shown that combining e-health with personal contact is more effective in realizing lifestyle behavior change [22,23].

Fourth, in this study, a mixed-meal challenge test was part of the baseline assessment and used as the basis for the personalized nutrition program. As this challenge test simulates consumption of a real meal and allows data capture on the postprandial state, it provides a more holistic view of the metabolic health status of an individual as compared to fasting measurements only [35,110]. A recent study by Berry et al. also showed the importance of postprandial measurements and the differences in postprandial glucose and lipid response to food between individuals [111].

Fifth, participants were offered personalized meals on weekdays for breakfast and lunch, whilst most personalized nutrition studies only offer recommendations and not the actual foods. This makes it easy to adhere to the personalized nutrition recommendations, at least during breakfast and lunch.

Last, the focus in this study was on the quality of the provided meals (ingredients, macronutrient quality, micronutrient content) and not the quantity of meals. The caloric content of meals was equal for all participants. Therefore, the results from this study showed the added value of a high-quality diet and not merely the effects of caloric restriction. Calorie intake did decrease during the intervention, but this was likely the result of the higher satiating properties of the healthy personalized foods.

## 5. Conclusions

In our study, we have shown that a PSN program on a workforce has positive effects on health behavior, body composition and markers of health status for groups A and G (as other groups were underrepresented in the study), thus showing that PSN programs can improve health outcomes. Our study suggests that personalized nutrition may enable changes in dietary intakes that have not occurred through public health recommendations, for example, the recommendation to reduce sodium intake by 20% [112]. Additionally, between-group differences indicate that personalized dietary programs may be an effective approach in realizing targeted behavior change in specific health-compromised individuals or target groups. Considering these two aspects, the possibility exists that in the future, personalized nutrition may provide the tools and motivation to enable individuals to achieve recommendations and reduce the health and economic burden of chronic diseases.

## Figures and Tables

**Table 1:** Biological factors and cut-off values used to generate personalized recommendations ^1^.

Advice Category	Personalized Advice	Personalization Factor ^2^	Classification	Personalization Based on SNP
Energy intake advice	Caloric intake	body weight, height, age, gender, physical activity	Mifflin St. Jeor equation: BMR (kcal/day) = 10 × weight (kg) + 6.25 × height (cm) − 5 × age (y) + a_1_ (kcal/day), a_1_ = +5 for males and −161 for females. Total energy expenditure = BMR × daily PAL Total PAL = PAL daily pattern + PAL sport/leisure_1_ + … + PAL sport/leisure_n_	-
Personalized Diet Types & SNP-based macronutrient advice	Protein intake	glucose tolerance, disposition index ^3^, blood-pressure	normal = normal, glucose intolerance = IFG, IGT, IFG/IGT or T2D low disposition index (<1.5), normal disposition index (>1.5) optimal (SBP <120 and DBP <80 mmHg), elevated (SBP ≥120 or DBP ≥80 mmHg)	**FTO rs9939609^5^**
	Carbohydrate intake	waist circumference, 2-h glucose	normal (M ≤ 40-inch, F ≤ 35 in), elevated (M > 40-in, F > 35 in) normal (<7.77 mmol/L; <140 mg/dL); elevated (≥7.77 mmol/L; ≥140 mg/dL)	**FTO rs9939609^5^** ADAMTS9 rs4607103 GCKR rs780094
	Fat intake	waist circumference, blood pressure, LDL cholesterol	normal (M ≤ 40 in, F ≤ 35 in), elevated (M > 40 in, F > 35 in) optimal (SBP < 120 and DBP < 80 mmHg), elevated (SBP ≥ 120 or DBP ≥ 80 mmHg) normal (≤3.36 mmol/L; ≤130 mg/dL); elevated (>3.36 mmol/L; >130 mg/dL)	**FTO rs9939609^5^** FTO rs1121980 TCF7L2 rs7903146 FADS1 rs174546 FADS1 rs174548 IGF2BP2 rs4402960 PPARG rs1801282
Micronutrient advice	Fiber intake	fasting glucose, 2-h glucose, LDL cholesterol, blood pressure, waist circumference	normal (<5.55 mmol/L; <100 mg/dL); elevated (≥5.55 mmol/L; ≥100 mg/dL) normal (<7.77 mmol/L; <140 mg/dL); elevated (≥7.77 mmol/L; ≥140 mg/dL) optimal (<2.59 mmol/L; <100 mg/dL); increased (≥2.59 mmol/L; ≥100 mg/dL) optimal (SBP < 120 and DBP < 80 mmHg), elevated (SBP ≥ 120 or DBP ≥ 80 mmHg) normal (M ≤ 40 in, F ≤ 35 in), elevated (M > 40 in, F > 35 in)	ADAMTS9 rs4607103 TCF7L2 rs7903146
	MUFA intake	disposition index ^3^ LDL cholesterol, blood pressure, fasting TG, postprandial TG ^4^,	low disposition index (<1.5), normal disposition index (>1.5) normal (≤3.36 mmol/L; ≤130 mg/dL); elevated (>3.36 mmol/L; >130 mg/dL) optimal (SBP <120 and DBP <80 mmHg), elevated (SBP ≥120 or DBP ≥80 mmHg) normal (≤1.7 mmol/L; ≤150 mg/dL); elevated (>1.7 mmol/L; >150 mg/dL) normal (≤2.5 mmol/L); elevated (>2.5 mmol/L)	-
	Omega-3 intake	blood pressure, fasting TG, postprandial TG ^4^, omega-3 index	optimal (SBP <120 and DBP <80 mmHg), elevated (SBP ≥120 or DBP ≥80 mmHg) normal (≤1.7 mmol/L; ≤150 mg/dL); elevated (>1.7 mmol/L; >150 mg/dL) normal (≤2.5 mmol/L); elevated (>2.5 mmol/L) optimal (>8 %); intermediate or low (≤8 %)	**FADS1 rs174546^5^** **FADS1 rs174548^5^**
	Phytosterols	LDL cholesterol	optimal (<2.59 mmol/L); increased (≥2.59-≤3.36 mmol/L); elevated (>3.36 mmol/L)	-
	Vitamin C intake	blood pressure, age, gender	optimal (SBP <120 and DBP <80 mmHg), elevated (SBP ≥120 or DBP ≥80 mmHg)	-
	Vitamin B2 intake	blood pressure, age, gender	optimal (SBP <120 and DBP <80 mmHg), elevated (SBP ≥120 or DBP ≥80 mmHg)	**MTHFR rs1801133^5^**
SNP-based narratives	Physical activity	-		ACTN3 rs1815739 FTO rs1121980
	Vitamin D	-		GC rs7041
		-		GC rs4588
		-		GC rs2282679
	Lactose intolerance	-		MCM6 rs182549
		-		MCM6 rs4988235
	Caffeine sensitivity	-		CYP1A2 rs762551
	Salt sensitivity	-		AGT rs5051
		-		AGT rs699

Abbreviations: BMR, basal metabolic rate; BW, body weight; DBP, diastolic blood pressure; F, females; IFG, impaired fasting glucose; IGT, impaired glucose tolerance; LDL, low density lipoprotein; M, Males; PAL, physical activity level; SBP, systolic blood pressure SNP, single nucleotide polymorphism; T2D, type 2 diabetes; TG, triglycerides. ^1^ This table is a simplified representation of the algorithms used for personalized advice; the actual algorithms are more complex and contain interdependencies; only a few micronutrient examples are included for illustration. Macro- and micronutrient recommendations were used to drive personalized recipes and meals. Complete algorithms and decision trees can be requested from the authors. ^2^ Personalization factors are variables that are used to drive personalized recommendations; these include demographics, anthropometrics and blood biomarkers. ^3^ Disposition index is calculated from glucose and insulin response curves after the challenge beverage consumption.^4^ Postprandial markers were measured at 30 and 120 min after challenge beverage consumption. ^5^ SNPs indicated in bold drove personalized dietary recommendations if the risk-variants of these SNPs coincided with an unhealthy phenotype; all other SNPs were only used to help provide additional context and supporting recommendations.

**Table 2:** Macronutrient ranges and target for dietary programs for the personalized diet types (PDTs).

	Carbohydrates	Fat	Protein
PDT	% of Total Energy (Target %)		
A	45–65 (50)	20–40 (30)	10–22 (20)
B	45–65 (60)	20–30 (20)	10–22 (20)
C	35–50 (45)	20–40 (40)	10–22 (15)
D	45–65 (45)	20–40 (25)	18–35 (30)
E	45–65 (45)	20–30 (20)	18–35 (35)
F	35–50 (35)	20–40 (30)	18–35 (35)
G	35–50 (40)	20–30 (25)	18–35 (35)

**Table 3:** Descriptive statistics for the per protocol (PP) and subgroup populations (group A, group G) at inclusion (week 0).

Variable	PP (*n* = 73)		Group A (*n* = 48)		Group G (*n* = 22)	
	Mean	SD	Mean	SD	Mean	SD
Gender (*n*, men/women)	25/48		15/33		9/13	
Age (years) **	43.1	8.7	40.9	8.1	47.8	8.3
Anthropometrics and Vitals						
BMI (kg/m^2^) ***	27.4	4.0	26.0	3.3	30.5	3.6
Body weight (kg) **	77.8	15.5	72.6	13.2	89.4	14.6
Body fat (%) ****	32.0	7.6	30.3	6.8	36.8	7.1
Muscle mass (kg)	50.1	10.7	48.3	9.9	53.7	11.8
Waist circumference (cm) ****	94.6	13.0	89.7	10.7	105.7	10.7
Hip circumference (cm) **	104.8	10.1	102.4	8.4	111.9	7.0
Systolic blood pressure (mmHg)	119.2	16.4	116.6	16.1	123.1	16.4
Diastolic blood pressure (mmHg)	73.7	8.7	72.2	8.0	75.6	8.6
Clinical Chemistry (fasting)						
C-peptide (nmol/L)	0.48	0.20	0.43	0.15	0.54	0.21
Glucose (mmol/L)	4.41	0.47	4.30	0.44	4.64	0.47
HDL (mmol/L)	1.52	0.40	1.60	0.43	1.39	0.27
LDL (mmol/L)	2.62	0.54	2.54	0.47	2.78	0.65
Total cholesterol (mmol/L)	4.68	0.66	4.66	0.62	4.70	0.75
Triglycerides (mmol/L)	1.15	0.57	1.11	0.61	1.25	0.51
Indices						
HOMA-IR	0.094	0.043	0.082	0.029	0.119	0.057
Matsuda index	212.0	82.4	230.8	77.5	176.2	79.0

Abbreviations: BMI, body mass index; HDL, high-density lipoprotein; HOMA-IR, Homeostatic Model Assessment for Insulin Resistance; LDL, low-density lipoprotein; *n*, number of observations PP, per protocol; SD, standard deviation. Statistically significant differences between group A and group G at baseline are noted (** *p* < 0.01; *** *p* < 0.001; **** *p* < 0.0001).

**Table 4:** Descriptive statistics of lifestyle factors during the intervention period (weeks 10 to 20).

	Week 10: Mean (SD)		Week 20: Mean (SD)		Difference:	
					Week 20–Week 10 (%) ^1^	
	A (*n* = 48)	G (*n* = 22)	A (*n* = 48)	G (*n* = 22)	A (*n* = 48)	G (*n* = 22)
Macronutrient intake ^1^						
Calories (kcal) ***	1877.1 (554.4)	1974.0 (520.0)	1681.5 (490.6)	1613.5 (324.5)	−10.40%	−18.30%
Carbohydrates (g) **	189.9 (64.9)	217.4 (83.1)	182.0 (58.3)	163.6 (43.5)	−4.20%	−24.70%
Carbohydrates (en%)	40.6 (7.2)	43.4 (5.4)	43 (6.6)	40.9 (8.5)	6%	−5.80%
Protein (g)	87.9 (24.8)	82.7 (16.2)	79.9 (24.6)	88.8 (27.6)	−9.10%	7.40%
Protein (en%)	19.1 (3.8)	17.3 (3.5)	19.9 (4.3)	22.0 (5.5)	4.10%	27.7% **
Fat (g) ****	80.2 (27.7)	82.6 (21.2)	65.0 (22.2)	62.4 (16.8)	−18.90%	−24.70%
Fat (en%) **	38.2 (5.4)	37.9 (5.8)	34.6 (6.2)	34.7 (5.0)	−9.50%	−8.50%
SFA (g) ***	25.8 (10.3)	25.2 (7.1)	20.6 (7.2)	18.8 (6.6)	−20.30%	−25.50%
SFA (en%)	12.3 (3.0)	11.7 (3.0)	11.0 (2.6)	10.4 (2.3)	−10.60%	−11.70%
PUFA (g) **	12.4 (5.9)	11.7 (5.5)	9.6 (4.7)	9.3 (4.3)	−22.60%	−20.50%
PUFA (en%)	6.0 (2.3)	5.4 (2.5)	5.2 (2.0)	5.1 (2.0)	−12.60%	−5.60%
MUFA (g)	22.0 (10.6)	20.4 (10.8)	17.8 (8.6)	17.0 (8.0)	−19.10%	−16.80%
MUFA (en%)	10.5 (3.5)	9.2 (3.6)	9.7 (3.4)	9.4 (3.3)	−8.50%	1.60%
Total sugar (g) ****	63.9 (33.5)	82.7 (40.5)	55.8 (30.2)	55.6 (23.9)	−12.70%	−32.70%
Total sugar (en%)	13.7 (5.7)	16.3 (5.1)	13.3 (5.4)	13.8 (5.4)	−3.90%	−15.70%
Total fiber (g) ^2^	17.3 (5.6)	17.6 (8.0)	19.0 (6.1)	17.8 (5.9)	9.70%	1.60%
Total fiber (en%) ^2^ ****	1.9 (0.6)	1.8 (0.6)	2.4 (0.8)	2.2 (0.7)	25.50%	24.70%
Micronutrient intake ^1^						
Sodium (mg) ***	2799.6 (895.5)	2795.3 (885.2)	2212.8 (892.3)	2371.7 (829.7)	−21%	−15.20%
Potassium (mg) *	1983.7 (781.0)	1777.7 (714.6)	2241.1 (716.4)	2233.8 (831.9)	13%	59.40%
Magnesium (mg) **	187.3 (66.7)	222.3 (150.5)	238.5 (82.5)	257.9 (78.2)	27.30%	15.90%
Vitamin C (mg) ***	74.4 (55.2)	72.3 (39.1)	106.1 (67.3)	111.8 (58.4)	42.60%	54.50%
Beta-carotene (mcg)	3074.0 (4330.5)	1534.2 (2740.1)	3415.7 (2518.0)	5970.8 (4316.1)	11.10%	289.2% ****
Physical activity ^3^						
Resting heart rate (bpm) ****	63.4 (6.9)	66.0 (7.1)	62.4 (6.7)	63.6 (7.0)	−1.70%	−2.90%
Steps (*n*/day) ****	9319 (3073)	8558 (1856)	10234 (3206)	8957 (1865)	8.50%	6.50%
Sleep ^3^						
Sleep (h/day)	7.3 (0.7)	6.9 (1.1)	7.1 (1.2)	7.0 (1.1)	−2.80%	1.40%

Abbreviations: bpm, beats per minute; en, energy; MUFA, monounsaturated fatty acid; *n*, number of observations; PP, per protocol; PUFA, polyunsaturated fatty acids; SD, standard deviation; SFA, saturated fatty acids. ^1^ Statistically significant differences are noted in the last two columns for changes in groups A or G, respectively, and in the first column for changes in the PP population (* *p* < 0.05; ** *p* < 0.01; *** *p* < 0.001; **** *p* < 0.0001). ^2^ The ‘TotalFiber_2016_p’ variable was used as the time of analysis was considered a more reliable indicator of fiber intake than the ‘post-2016 fiber’ variable (Food Processor Nutrition Analysis Software, ESHA, Salem, OR, USA). The ESHA database is built based on food labels and restaurant labeling as well as the USDA database. The FDA change in fiber qualifications has not fully translated into the “post-2016 fiber‘ variable. ^3^ Physical activity and sleep differences were considered statistically significant at *p* < 0.01.

**Table 5:** Descriptive statistics of anthropometrics, vitals, clinical chemistry and indices values during the intervention period (weeks 10 to 20).

Variable ^1^	Week 10: Mean (SD)		Week 20: Mean (SD)		Difference: Week 20–Week 10 (%) ^1^	
	A (*n* = 48)	G (*n* = 22)	A (*n* = 48)	G (*n* = 22)	A (*n* = 48)	G (*n* = 22)
Anthropometrics and Vitals						
BMI (kg/m^2^) ****	26.0 (3.4)	30.7 (3.8)	25.7 (3.3)	29.9 (3.8)	−1.20%	−2.60%
Body weight (kg)	73.0 (13.4)	90.0 (15.0)	72.1 (13.3)	89.0 (15.4)	−1.2% **	−1.1% ****
Body fat (%) *	30.5 (7.1)	36.2 (6.0)	29.8 (6.9)	35.9 (6.7)	−2.30%	−0.90%
Muscle mass (kg)	48.3 (9.7)	54.2 (11.6)	48.1 (10.0)	54.4 (11.7)	−0.50%	0.40%
Waist circumference (cm)	89.5 (11.0)	104.5 (11.8)	89.0 (9.5)	104.3 (11.8)	−0.60%	−0.20%
Hip circumference (cm) ****	101.2 (8.4)	111.5 (7.3)	99.6 (8.4)	108.0 (7.8)	−1.60%	−3.10%
Systolic BP (mmHg)	118.0 (13.8)	122.8 (15.8)	114.7 (15.0)	119.2 (14.3)	−2.80%	−2.90%
Diastolic BP (mmHg)	73.8 (7.7)	76.5 (8.2)	70.9 (7.1)	74.8 (9.1)	−3.90%	−2.20%
Clinical Chemistry (fasting)						
C-peptide fasting (nmol/L)	0.54 (0.24)	0.68 (0.23)	0.47 (0.19)	0.66 (0.25)	−12.10%	−2.70%
C-peptide 2 h (nmol/L)	1.39 (0.69)	1.91 (0.73)	1.42 (0.79)	1.73 (0.69)	1.90%	−9.70%
Glucose fasting (mmol/L)	4.48 (0.47)	4.75 (0.41)	4.68 (0.51)	5.18 (0.49)	4.50%	9.00%
Glucose 2 h (mmol/L)	5.37 (0.83)	5.94 (0.87)	5.56 (0.72)	6.10 (0.63)	3.60%	2.70%
HDL cholesterol (mmol/L)	1.54 (0.39)	1.38 (0.40)	1.62 (0.40)	1.28 (0.36)	5.10%	−6.80%
LDL cholesterol (mmol/L)	2.67 (0.53)	2.88 (0.64)	2.44 (0.50)	2.44 (0.40)	−8.40%	−15.4% **
Total cholesterol (mmol/L)	4.70 (0.60)	4.96 (0.74)	4.61 (0.61)	4.46 (0.61)	−2%	−9.9% ****
Triglycerides (mmol/L)	1.16 (0.61)	1.58 (0.56)	1.32 (1.0)	1.64 (0.47)	13.20%	3.70%
Indices						
HOMA-IR	0.108 (0.050)	0.142 (0.046)	0.100 (0.045)	0.154 (0.062)	−7.40%	8.50%
Matsuda index	202.8 (89.2)	129.9 (40.6)	191.1 (66.9)	125.8 (45.7)	−5.80%	−3.20%

Abbreviations: BP, blood pressure; HDL, high-density lipoprotein; HOMA-IR, Homeostatic Model Assessment for Insulin Resistance; LDL, low-density lipoprotein; *n*, number of observations; PP = per protocol; SD, standard deviation; ^1^ Statistically significant differences are noted in the last two columns for changes in groups A or G respectively and in the first column for changes in the PP population (* *p* < 0.05; ** *p* < 0.01; **** *p* < 0.0001).

## Data Availability

The data presented in this study are available on reasonable request from the corresponding author. The data are not publicly available due to privacy reasons.

## Supplementary Materials

The following are available online at https://www.mdpi.com/article/10.3390/nu13061763/s1, Table S1: Examples of SNP-based narratives; Table S2: Lifestyle factors, anthropometrics, vitals, clinical chemistry and indices at baseline (week 0), after run-in (week 10), and after the intervention period (week 20) for the PP population.
