# Supplementary material for: A Novel Personalized Systems Nutrition Program Improves Dietary Patterns, Lifestyle Behaviors and Health-Related Outcomes: Results from the Habit Study
Source: Nutrients. 2021 May 22;13(6):1763. doi: 10.3390/nu13061763 (PMC8224682; doi:10.3390/nu13061763)
Supplement: Supplementary file 1 [file nutrients-13-01763-s001.zip › nutrients-1185002-supplementary.pdf]

Table S1. Examples of SNP-based narratives.

SNP	Narrative	Ref.
FADS1 rs174546 and rs174548	FADS1 is the name of a gene in your body. The product of this gene is the fatty acid desaturase (FADS1) protein, which plays a role in regulating the synthesis of fatty acids. One version, or "variant" of this gene called FADS1 rs174546/rs174548 is associated with slower production of omega-3 fatty acids and other important polyunsaturated fatty acids. Eating long chain omega-3 polyunsaturated fats, such as those found in fatty fish like salmon, may help reduce the effect of this variant.<br><br>*"Impact" result:*<br>You have the FADS1 rs174546/rs174548 risk variant that is associated with slower production of omega-3s and other healthy fatty acids.<br><br>*Non-impact:*<br>You do not have the FADS1 rs174546/rs174548 risk variant that is associated with slower production of omega-3s and other healthy fatty acids.	1-3
MCM6 rs182549 and rs4988235	MCM6 is the name of a gene in your body. The product of this gene affects the production of lactase, an enzyme that breaks down lactose, the type of sugar in dairy products such as milk and cheese. One version, or "variant" of this gene called MCM6 rs182579/rs4988235 may affect your ability to digest lactose. If you experience complaints, limiting the amount of dairy in the diet may reduce the effects of this variant.<br><br>*"Impact" (non-green) result:*<br>You have the MCM6 rs182579/rs4988235 risk variant that may affect your ability to digest lactose.<br><br>*Non-impact:*<br>You do not have the MCM6 rs182579/rs4988235 risk variant that may affect your ability to digest lactose.	4-5
IGF2BP2 rs4402960	IGF2BP2 is the name of a gene in your body. The product of this gene is the insulin-like growth factor binding protein which is important for the production of IGF2, a hormone that is structurally similar to insulin. One version, or "variant" of this gene called IGF2BP2 rs4402960 may affect the function of your insulin producing cells and as a result how your body produces insulin and uses glucose. A lower-fat diet may help reduce the effect of this variant.<br><br>*"Impact" (non-green) result:*<br>You have the IGF2BP2 rs4402960 risk variant that may affect the function of your insulin producing cells and as a result how your body produces insulin and uses glucose.<br>*Non-impact:*<br><br>*Non-impact:*<br>You do not have the IGF2BP2 rs4402960 risk variant that may affect the function of your insulin producing cells and as a result how your body produces insulin and uses glucose.	6

1. Bokor S, Dumont J, Spinneker A, Gonzalez-Gross M, Nova E, Widhalm K, Moschonis G, Stehle P, Amouyel P, De Henauw S, Molnàr D, Moreno LA, Meirhaeghe A, Dallongeville J; HELENA Study Group. Single nucleotide polymorphisms in the FADS gene cluster are associated with delta-5 and delta-6 desaturase activities estimated by serum fatty acid ratios. J Lipid Res. 2010 Aug;51(8):2325-33. doi: 10.1194/jlr.M006205. Epub 2010 Apr 28. PMID: 20427696; PMCID: PMC2903808.
2. Coltell O, Sorlí JV, Asensio EM, Barragán R, González JI, Giménez-Alba IM, Zanón-Moreno V, Estruch R, Ramírez-Sabio JB, Pascual EC, Ortega-Azorín C, Ordovas JM, Corella D. Genome-Wide Association Study for Serum Omega-3 and Omega-6 Polyunsaturated Fatty Acids: Exploratory Analysis of the Sex-Specific Effects and Dietary Modulation in Mediterranean Subjects with Metabolic Syndrome. Nutrients. 2020 Jan 24;12(2):310. doi: 10.3390/nu12020310. PMID: 31991592; PMCID: PMC7071282.
3. O'Neill CM, Minihane AM. The impact of fatty acid desaturase genotype on fatty acid status and cardiovascular health in adults. Proc Nutr Soc. 2017 Feb;76(1):64-75. doi: 10.1017/S0029665116000732. Epub 2016 Aug 16. PMID: 27527582.
4. Enattah NS, Sahi T, Savilahti E, Terwilliger JD, Peltonen L, Järvelä I. Identification of a variant associated with adult-type hypolactasia. Nat Genet. 2002 Feb;30(2):233-7. doi: 10.1038/ng826. Epub 2002 Jan 14. PMID: 11788828.
5. Bersaglieri T, Sabeti PC, Patterson N, Vanderploeg T, Schaffner SF, Drake JA, Rhodes M, Reich DE, Hirschhorn JN. Genetic signatures of strong recent positive selection at the lactase gene. Am J Hum Genet. 2004 Jun;74(6):1111-20. doi: 10.1086/421051. Epub 2004 Apr 26. PMID: 15114531; PMCID: PMC1182075.
6. Ruchat SM, Elks CE, Loos RJ, Vohl MC, Weisnagel SJ, Rankinen T, Bouchard C, Pérusse L. Evidence of interaction between type 2 diabetes susceptibility genes and dietary fat intake for adiposity and glucose homeostasis-related phenotypes. J Nutrigenet Nutrigenomics. 2009;2(4-5):225-34. doi: 10.1159/000259341. Epub 2010 Mar 9. PMID: 20215779.

Table S2. Lifestyle factors, anthropometrics, vitals, clinical chemistry and indices at baseline (Week 0), after run-in (Week 10), and after intervention period (week 20) for the PP population.

Variable	PP Population at run-in (week 0 vs week 10)				
	Week 0 (SD)	Week 10 (SD)	Week 20 (SD)	Difference wk 0-10[1] (%)	Difference wk 10-20[1] (%)
**Macronutrient intake[1]**					
Calories (kcal)	2319 (2688)	1906 (537)	1650 (461)	5.3	-13.4 ***
Carbohydrates (g)	194.3 (68.8)	197.9 (70.6)	175.7 (56.0)	1.8	-11.2 **
Carbohydrates (en%)	42.9 (8.5)	41.4 (6.7)	42.6 (7.2)	-3.4	2.9
Protein (g)	87.5 (64.8)	86.4 (22.6)	83.1 (25.4)	-1.3	-3.8
Protein (en%)	19.2 (7.6)	18.5 (3.8)	20.5 (4.7)	-3.2	10.6
Fat (g)	73.6 (25.6)	81.1 (25.5)	63.8 (21.5)	10.2 *	-21.3 ****
Fat (en%)	36.7 (6.6)	38.2 (5.4)	34.6 (5.7)	4.0	-9.4 **
SFA (g)	25.1 (18.9)	25.6 (9.2)	19.7 (7.2)	2.1	-22.9 ***
SFA (en%)	12.0 (4.8)	12.1 (2.9)	10.7 (2.5)	0.4	-11.4
PUFA (g)	9.6 (6.5)	12.1 (5.7)	9.6 (4.5)	26.0 **	-21.2 **
PUFA (en%)	4.7 (2.3)	5.8 (2.3)	5.2 (2.0)	21.7	-10.1
MUFA (g)	17.1 (10.0)	21.4 (10.5)	17.6 (8.5)	24.8 **	-17.6
Monounsaturated fat (en%)	8.7 (4.9)	10.1 (3.5)	9.6 (3.3)	15.5 *	-4.7
Total sugar (g)	68.1 (32.0)	69.5 (36.0)	56.5 (27.7)	2.1	-18.7 ****
Total sugar (en%)	15.2 (5.2)	14.5 (5.6)	13.6 (5.3)	-4.2	-6.7

Total fiber (g)[2]	22.4 (36.7)	17.4 (6.4)	19.1 (6.3)	-22.0	9.1
Total fiber (en%)[2]	2.3 (2.2)	1.9 (0.6)	2.4 (0.8)	-18.6	26.8 ****
**Micronutrient intake[1]**					
Sodium (mg)	2971 (1109)	2825 (908)	2279 (886)	-4.9	-19.3 ***
Potassium (mg)	1693 (665)	1912 (756)	2240 (771)	13.0	17.1 *
Magnesium (mg)	173.2 (77.2)	197.2 (98.9)	245.0 (85.5)	13.9	24.2 **
Vitamin C (mg)	79.5 (51.0)	74.2 (50.9)	107.7 (62.5)	-6.7	45.3 ***
Beta-carotene (mcg)	2319 (2688)	2701 (3965)	4382 (3470)	16.5 ***G*	62.2
**Physical activity & sleep[3]**					
Resting heart rate (bpm)	n/a	64.4 (7.4)	62.9 (7.1)	n/a	-1.3 ****
Steps (nr/day)	n/a	9015 (2749)	9803 (2915)	n/a	727 ****
Sleep (h/day)	n/a	7.1 (1.1)	6.9 (1.4)	n/a	-0.2
**Anthropometrics and Vitals[1]**					
BMI (kg/m2)	27.4 (4.0)	27.6 (4.2)	26.8 (3.8)	0.8	-2.8****
Body weight (kg)	77.8 (15.5)	78.2 (15.9)	76.5 (15.6)	0.6	-2.2
Body fat (%)	32.0 (7.6)	32.4 (7.8)	31.2 (7.4)	1.4	-3.6 *
Muscle Mass (kg)	50.1 (10.7)	50.2 (10.6)	49.8 (10.7)	0.1	-0.7
Waist Circumference (cm)	94.6 (13.0)	94.0 (13.3)	93.1 (12.1)	-0.6	-1.0
Hip Circumference (cm)	104.8 (10.1)	104.1 (9.9)	101.7 (8.9)	-0.7	-2.2 ****
Systolic BP (mmHg)	119.2 (16.4)	119.7 (14.3)	116.2 (14.6)	0.4	-3.0
Diastolic BP (mmHg)	73.7 (8.7)	74.8 (7.9)	72.5 (8.2)	1.4	-3.1

**Clinical Chemistry (fasting)**[1]					
C-peptide fasting (nmol/l)	0.48 (0.20)	0.58 (0.24)	0.52 (0.22)	22.7 **	-10.9
C-peptide 2h (nmol/l)	1.29 (0.65)	1.54 (0.73)	1.45 (0.77)	19.4 **	-6.1
Glucose fasting (mmol/l)	4.41 (0.47)	4.57 (0.48)	4.83 (0.54)	3.4	5.7
Glucose 2h (mmol/l)	5.23 (1.01)	5.54 (0.88)	5.69 (0.80)	5.9 *	2.7
HDL cholesterol (mmol/l)	1.52 (0.40)	1.47 (0.42)	1.50 (0.44)	-3.8	2.4
LDL cholesterol (mmol/l)	2.62 (0.54)	2.70 (0.64)	2.44 (0.47)	2.8	-9.4
Total cholesterol (mmol/l)	4.68 (0.66)	4.73 (0.75)	4.56 (0.61)	1.0	-3.6
Triglycerides (mmol/l)	1.15 (0.57)	1.28 (0.62)	1.38 (0.88)	11.0	8.1
**Indices**[1]					
HOMA-IR	0.094 (0.043)	0.119 (0.050)	0.114 (0.051)	26.4 **	-4.1
Matsuda Index	212.0 (82.4)	179.3 (83.3)	174.8 (68.1)	-15.4 ***	-2.5

Abbreviations: BP, blood pressure; BMI, body mass index; bpm, beats per minute; en, energy; HDL, high-density lipoprotein; HOMA-IR, Homeostatic Model Assessment for Insulin Resistance; LDL, low-density lipoprotein; MUFA, monounsaturated fatty acid; n, number of observations; PP, per protocol; PUFA, polyunsaturated fatty acids; SD, standard deviation; SFA, saturated fatty acids.

[1]Statistically significant differences in the PP population are noted in the last two columns for changes from baseline to run-in and run-in to end of intervention respectively (* p<0.05; ** p<0.01; *** p<0.001; **** p<0.0001); G, only for group G, but not for group A.

[2]The 'TotalFiber_2016_p' variable was used as the time of analysis it was considered a more reliable indicator of fiber intake than the 'post 2016 fiber' variable (Food Processor Nutrition Analysis Software, ESHA, Salem, OR). The ESHA database is built based on food labels and restaurant labeling as well as the USDA database. The FDA change in fiber qualifications has not fully translated into the "post 2016 fiber' variable.

[3]Physical activity and sleep differences were considered statistically significant at p<0.01.
